# Advances in α-Arylation of Carbonyl Compounds: Diaryliodonium Salts as Arylating Agents

**DOI:** 10.3390/molecules30143019

**Published:** 2025-07-18

**Authors:** Xiao-Wei Chen, Jia-Le Chen, Ling-Hui Zhang, Huhu Zhang, Xiaojun Chen, Xiaohui Fan

**Affiliations:** 1 School of Chemistry and Chemical Engineering, Lanzhou Jiaotong University, Lanzhou 730070, China; lzjtdxcjl@163.com (J.-L.C.); zhangh2026@163.com (H.Z.); 2 State Key Laboratory of Applied Organic Chemistry, College of Chemistry and Chemical Engineering, Lanzhou University, Lanzhou 730000, China; 120220905621@lzu.edu.cn; 3 Animal Husbandry and Veterinary Bureau of Anding District, Dingxi 743021, China

**Keywords:** diaryliodonium salts, α-arylation, carbonyl compounds, arylating agents

## Abstract

Diaryliodonium salts are an important part of hypervalent iodine chemistry, owing to their highly electrophilic character, non-toxicity, and air and moisture stability, have been identified as an important arylating agent. It has been widely applied in the synthesis of natural products, drugs, and bioactive molecules bearing active α-arylation carbonyl units. Within the domain of α-arylation of carbonyl compounds using diaryliodonium salts, there is a notable absence in the literature of a comprehensive compilation dedicated to exclusive arylation processes involving these compounds. In this review, we focus on the overview of the recent advancements in utilizing diaryliodonium salts for α-arylation reactions, encompassing both racemic and asymmetric approaches to various carbonyl compounds including ketones, esters, enolates, and amides. Furthermore, we discuss the unique advantages and inherent limitations of diaryliodonium salts as arylating agents, as well as the underexplored application potentials that warrant further investigation in this rapidly evolving field.

## 1. Introduction

**Figure 1:**
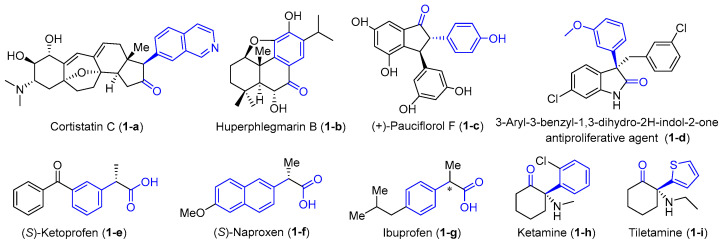
Selected natural products and bioactive molecules bearing an α-aryl carbonyl scaffold.

**Figure 2:**
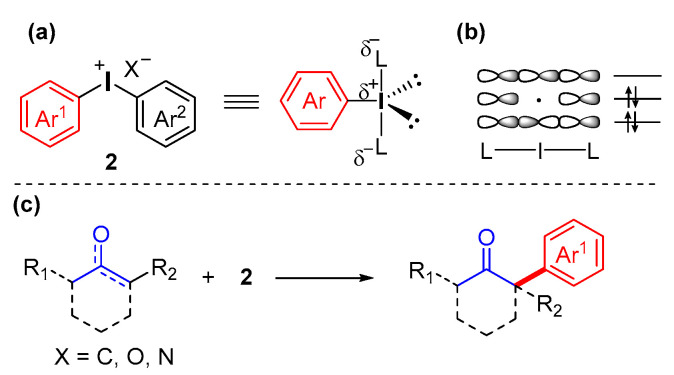
(**a**) General and T-shaped structure. (**b**) Orbitals in the hypervalent ω bond. (**c**) α-arylation of carbonyl compounds using diaryliodonium salts.

The α-aryl carbonyl compounds are prevalent in numerous natural products, bioactive molecules, and drugs of significance. Such scaffolds also serve as key building blocks in the total synthesis of biologically active molecules (Figure 1) [1,2,3,4,5,6,7,8,9,10]. Over the past few decades, numerous synthetic chemists, both domestically and internationally, have dedicated significant effort to the study of α-arylation of carbonyl groups. This research has given rise to a multitude of exceptional chemical synthesis methods and strategies [11,12,13,14,15,16] such as transition metal-catalyzed cross-coupling, transition metal-free α-arylation, metal-catalyzed photoredox reaction [17,18] and metal-free photoredox α-arylation [19]. According to the type of arylating agents employed, these agents can be classified into two major categories: (i) arylating nucleophilic, aryl metal, or boron species; (ii) arylating electrophiles, such as aryl halides, aryl triflates, diaryliodonium salts, etc. Among them, the diaryliodonium salt was first synthesized in 1894 by Meyer and Hartmann [20]. The structure features a trivalent iodine atom bonded to two aryl groups and one counter-anion. In the solid state, this compound adopts a three-coordinate, T-shaped geometry. Within this structure, the iodine atom forms a hypervalent, linear 3-center 4-electron bond with two of its substituents (Figure 2a).

This bonding imparts electrophilic character to the iodine center, resulting in excellent reactivity (Figure 2b). Consequently, a large variety of synthetic routes to diaryliodonium salts have been developed [21,22,23,24,25,26,27,28,29,30]. With the rapid development of the related synthetic methods, diaryliodonium salts have been widely recognized and utilized in the field of organic synthesis chemistry, such as metal-catalyzed cross-coupling, transition-metal-catalyzed C-H bond activation/aromatization, α-position arylation of carbonyl compounds, asymmetric tandem cyclization and arylation of heteroatoms [31,32,33,34,35,36,37,38,39,40,41,42,43,44,45,46,47,48,49], and photoredox catalysis involving diaryliodonium salts [50,51,52]. Meanwhile, these protocols have been successfully applied to the rapid synthesis of natural products, pharmaceutical candidates, treatment of cancer, oral topical analgesic (*S*)-Ketoprofen (**1-e**), oral non-steroidal anti-inflammatory drug (*S*)-Naproxen (**1-f**) and Ibuprofen (**1-g**), as well as clinical drugs ketamine (**1-h**) and tiletamine (**1-i**).

Over the past few decades, the α-arylation of carbonyl compounds is a significant area of research within this domain, with diaryliodonium salts emerging as efficient, mild, selective, and robust arylating agents to surmount this difficult problem. In 2022, Zhou and Yu [53] published a review article that covers the enantioselective α-arylation of carbonyl enolates and related compounds by iodonium salts. In 2025, Simlandy’s group [54] conducted a comprehensive review of metal-catalyzed enantioselective aryl transfer methodologies utilizing diaryliodonium salts. Despite significant achievements, to the best of our knowledge, there lacks a comprehensive review article to summarize the recent advances in the α-arylation of α-carbonyl compounds.

Within the domain of α-arylation of carbonyl compounds using diaryliodonium salts, there is a notable absence in the literature of a comprehensive compilation dedicated to exclusive arylation processes involving these compounds. In this review article, we summarize recent advances in the α-arylation of carbonyl compounds (such as ketones, esters, enolates, and amides, etc.) (Figure 2c).

## 2. The Racemic α-Arylation of Carbonyl Compounds

### 2.1. α-Arylation of Keto Carbonyl Compounds

In 1960, Yudis’ research group [55] reported the α-arylation of carbonyl compounds using diphenyliodonium salts as arylating agents. The reaction was conducted in *t*-BuOH using *t*-BuOK as base, wherein 1,3-dicarbonyls (**3**) (such as dimedone, dibenzoylmethane, and tribenzoylmethane) reacted with diphenylsulfonium salt (**2-a**) to afford α-phenylated ketones (**4-a**–**4-b**) and side products. Systematic screening of solvents and counter-anions identified *tert*-butanol and the iodide anion as optimal components.

**Scheme 1:**
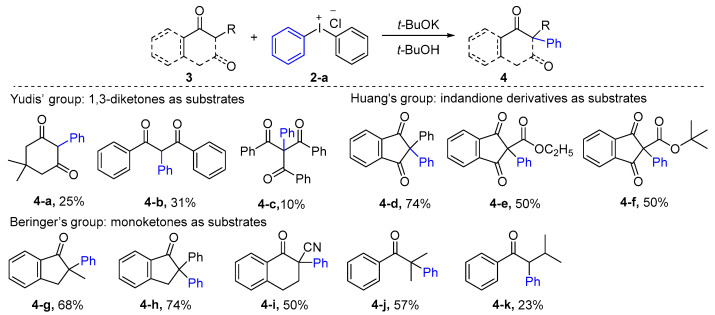
α-Phenylation of ketones.

In subsequent studies, Huang’s group [56] and Beringer’s group [57] employed the same reaction system to achieve the α-phenylation of indandione derivatives (**4-d**–**4-f**) and monoketones (**4-g**–**4-k**), respectively. The phenylated indandione derivatives product was obtained in moderate yield, whereas the phenylated monoketone product was afforded in moderate-to-good yield (Scheme 1).

**Scheme 2:**
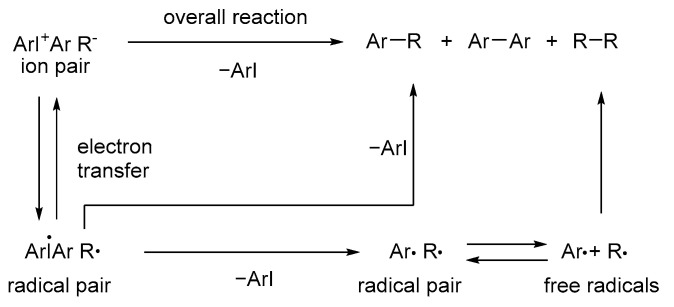
Proposed radical-pair mechanism.

Based on the observed reaction pathway, the group proposed a radical-pair mechanism: electron transfer from a carbanion (R^−^) to the iodonium ion (Ar–I^+^–Ar) generates transient radical pairs (R· and Ar·), which predominantly recombine via radical substitution on diphenyliodine or direct coupling to yield arylative products. Competing pathways involving radical dissociation generate side products (Ar-R, Ar-Ar, R-R) through stochastic recombination (Scheme 2). Furthermore, the byproduct observed in Beringer’s group’s study could also be explained by this reaction mechanism.

The synthesis of these arylation products demonstrates the utilization of diaryliodonium salts as arylating agents in organic synthesis. These experiments preliminarily investigated the reaction conditions, applicability, and mechanism of aryl iodonium salts as arylating reagents, laying the foundation for subsequent research on arylation.

**Scheme 3:**
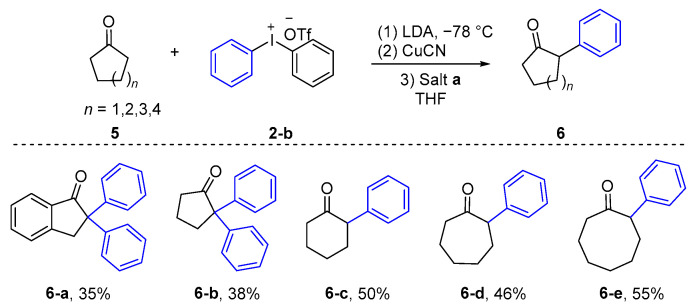
Cu-mediated α-phenylation of cyclic ketones.

In 1997, Ryan’s research group [58] reported the transition metal-mediated α-arylation of cyclic ketones using diaryliodonium triflates (Scheme 3). Employing CuCN as a catalyst, the reaction between diaryliodonium triflate (**2-b**) and cyclic ketones (**5**) selectively produced α-phenylated derivatives (**6**). Notably, 5-membered cyclic ketones exclusively underwent diarylation (**6-a**, **6-b**), while larger rings (6–8-membered) favored monoarylation (**6-c**, **6-d**, **6-e**.). This research group has developed efficient methods for synthesizing a wide range of stable diaryliodonium triflates and successfully applied them to the arylation of cyclic ketones. This work significantly expands both the synthetic methodologies for preparing diaryliodonium salts and their applicability as arylating agents.

**Scheme 4:**
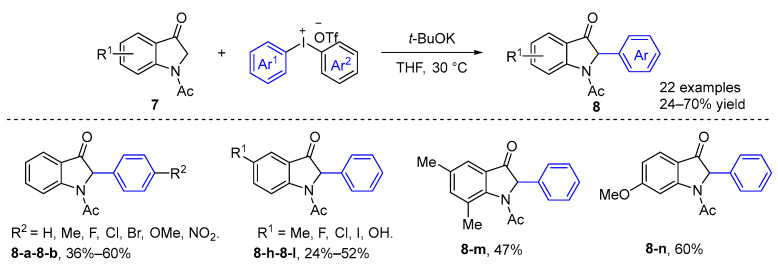
α-Arylation of indolone derivatives.

In 2015, Liu and co-workers [59] reported a *t*-BuOK-promoted method for the α-arylation of indolone derivatives (**7**) with diaryliodonium salts, achieving monoarylated indolone derivatives (**8**) in moderate-to-good yields (24–60%) (Scheme 4). This procedure demonstrated remarkable compatibility with a variety of functional groups and displayed a wide substrate applicability, showing adaptability to varying electronic and steric conditions. Moreover, transformation of monoarylated indolinones with diverse electrophiles has been demonstrated, enabling access to bioactive compounds featuring 2-arylated indolinone cores.

**Scheme 5:**
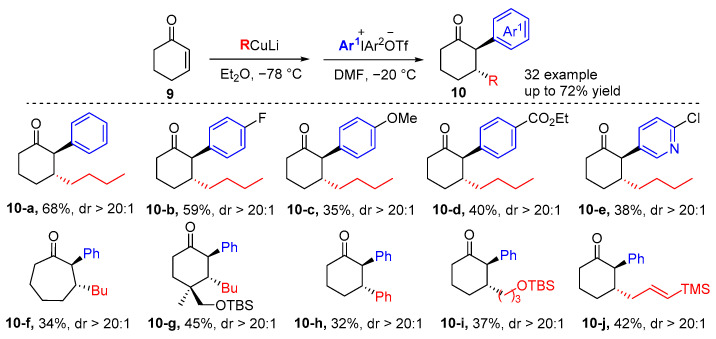
Cu-mediated one-pot Michael addition/α-arylation of an enone.

In 2015, Zhang’s research group [60] reported Cu-mediated one-pot Michael addition/α-arylation strategy to construct α-aryl-β-substituted cyclic ketone scaffolds (**10**) using cyclohex-2-en-1-one (**9**) as the substrate and diaryliodonium salts as arylating agents (Scheme 5). The transformation exhibits advantages including commercially accessible reagents, broad substrate tolerance, moderate-to-excellent yields, and high diastereoselectivity.

**Scheme 6:**
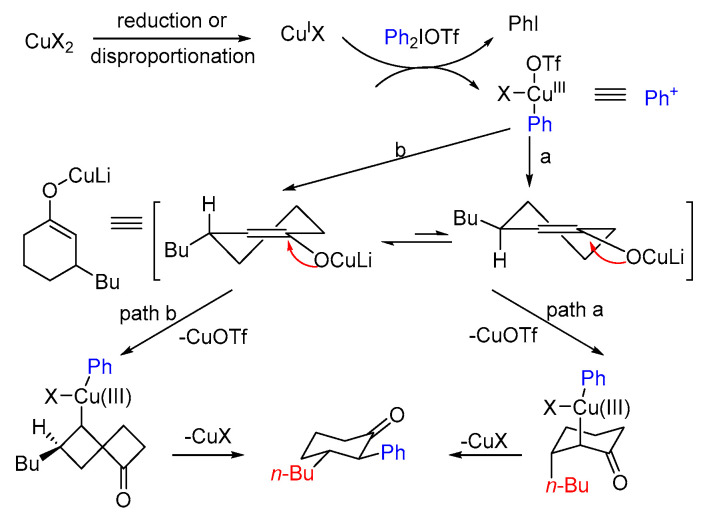
The mechanism of Cu-mediated one-pot Michael addition/α-arylation of an enone.

Based on the experimental results and previous studies, a possible mechanism for the current one-pot reaction was proposed (Scheme 6). Firstly, a catalytically active Cu(I) species—generated either in situ via formation of a copper enolate intermediate or derived from Cu(II) through reduction/disproportionation—reacts with the diaryliodonium salt to afford a key Cu(III)-aryl intermediate. This electrophilic Cu(III) species subsequently engages with the Michael addition-derived enolate intermediate through either path **a** or path **b**, followed by phenyl group transfer and C–C(Ar) bond formation via reductive elimination. This sequence delivers the product while regenerating the Cu(I) catalyst.

**Scheme 7:**
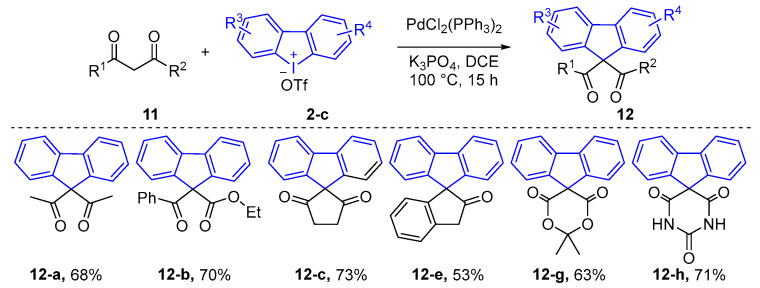
Synthesis of fluorene and spirofluorene derivatives.

In 2017, Wen and co-workers [61] reported a palladium-catalyzed dual C–C arylation reaction using cyclic diaryliodonium salts (**2-c**) and methylene-containing 1,3-dicarbonyl compounds (**11**) as substrates (Scheme 7). This method enabled the concise and efficient construction of structurally diverse fluorene derivatives (**12**) bearing all-carbon quaternary carbon centers. Moreover, the protocol was extended to synthesize novel pharmacologically active spirofluorene derivatives incorporating barbituric acid or indanone moieties, demonstrating distinctive synthetic value.

**Scheme 8:**
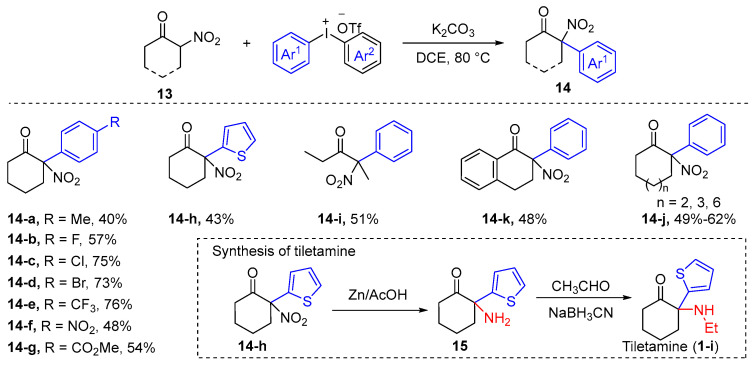
α-Arylation of α-nitro ketones and synthesis of tiletamine.

In 2019, Zhang’s research group [62] extended the frontiers of metal-free ketone functionalization by pioneering the transition metal-free α-arylation of α-nitro ketones (**13**) mediated by diaryliodonium salts. The protocol delivered structurally diverse α-aryl-α-nitro ketones (**14**) in synthetically viable yields (40–76%), while demonstrating exceptional functional group compatibility and extensive substrate generality (Scheme 8). Additionally, this group achieved a concise and efficient synthesis of the clinical drug Tiletamine (**1-i**) via this method through a three-step transformation with a 32% overall yield. In summary, the synthetic strategy developed by this research group not only features mild reaction conditions and broad substrate scope, but enables the production of synthetically valuable products.

**Scheme 9:**
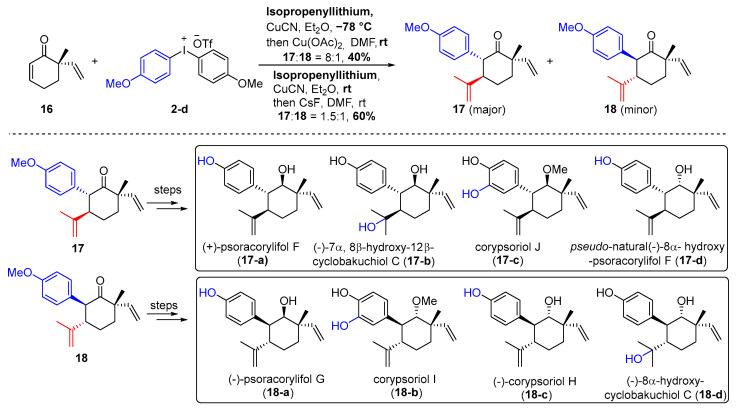
Total synthesis of several meroterpenoid natural products.

In 2023, our group [63] further achieved enantioselective divergent total syntheses of several meroterpenoids. (Scheme 9) The key transformations involved a one-pot Michael addition/α-arylation of an enone (**16**), utilizing diaryliodonium salts (**2-d**) as the arylating reagent to install the α-aryl group, yielding the desired arylation products (**17** and **18**). The authors have further expanded the use of diaryliodonium salts as arylation reagents in the total synthesis of meroterpenoid natural products.

### 2.2. α-Arylation of Ester Carbonyl Compounds

**Scheme 10:**
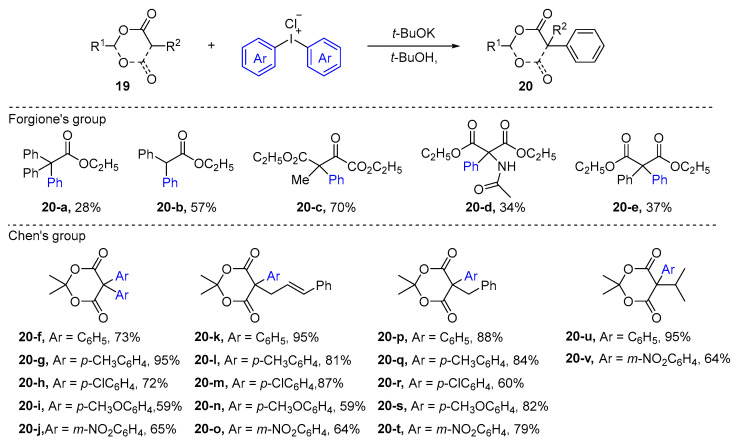
α-Phenylation of ester carbonyl compounds and isopropyl malonate.

In 1963, Forgione and co-workers [64] pioneered the α-phenylation of ester carbonyl compounds (**19**) using diphenyliodonium chloride as the arylating reagent. This transformation was achieved in a *t*-BuOK/*t*-BuOH system under reflux conditions, delivering α-arylated products (**20-a**–**20-e**) in modest yields (28–70%). They discovered that spatial steric hindrance significantly impacted the reaction. Furthermore, the benzhydrylation products, synthesized in an early exploratory phase without optimization, yielded suboptimally but are amenable to improvement. In 1987, Chen et al. [65] utilized the same reaction system to achieve the α-arylation of isopropylidene malonate and its 5-substituted derivates. The reaction afforded diarylated or monoarylated products (**20-f**–**20-v**) in good-to-excellent yields (Scheme 10).

A key feature of the above approach is that the α-arylated diesters can be hydrolyzed to α-aryl carboxylic acids or their esters. Therefore, this method provides an indirect yet efficient route for the preparation of α-aryl carboxylic acids or esters. Furthermore, the α-arylation of isopropylidene malonate and its 5-substituted derivatives proceeded in satisfactory yields regardless of whether the aryl ring bore electron-withdrawing groups (Cl, NO_2_) or electron-donating groups (such as CH_3_, OCH_3_).

**Scheme 11:**
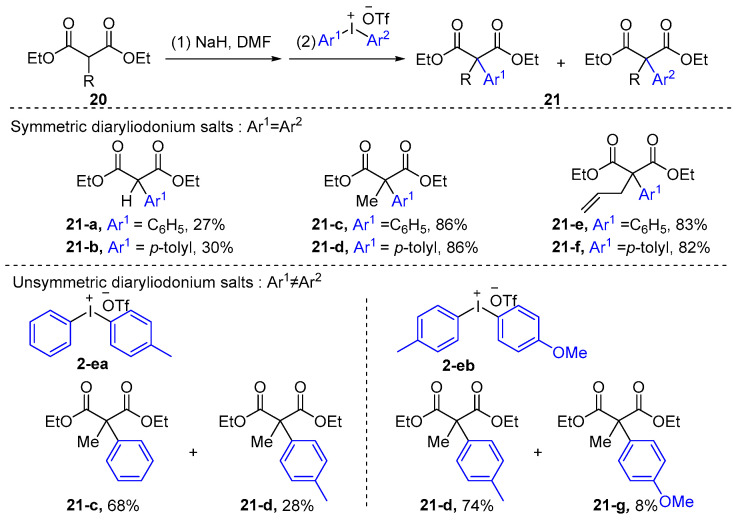
α-Arylation of β-ketoester.

In 1999, Chang and colleagues [66] reported the α-arylation of β-ketoester substrates (**20**) employing diaryliodonium salts as arylating agents under basic promotion (Scheme 11). The experimental results demonstrated that the corresponding aryl derivatives (**21**) were obtained in moderate-to-good yields under mild conditions. However, when Ar^1^ and Ar^2^ are distinct groups, the arylation reaction exhibits poor regioselectivity. This limitation significantly constrained the synthetic utility of unsymmetrical diaryliodonium salts. This challenge was subsequently addressed in later research.

**Scheme 12:**
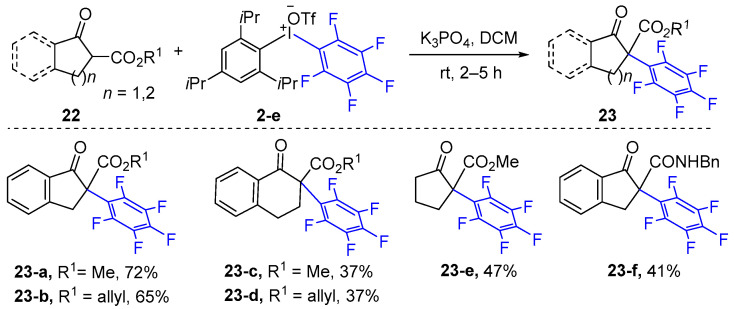
α-Pentafluorophenylation of β-ketoesters and β-ketoamides.

In 2014, Shibata and co-workers [67] synthesized a series of sterically hindered, unsymmetrical diaryliodonium salts, including pentafluorophenyl and triisopropylphenyl moieties (**2-f**). Employing these electrophilic aryl transfer reagents in conjunction with K_3_PO_4_ as the base, the group achieved α-pentafluorophenylation of β-ketoesters and β-ketoamides (**22**), affording α-pentafluorophenylated 1,3-dicarbonyl compounds featuring all-carbon quaternary stereocenters (**23**) (Scheme 12).

**Scheme 13:**
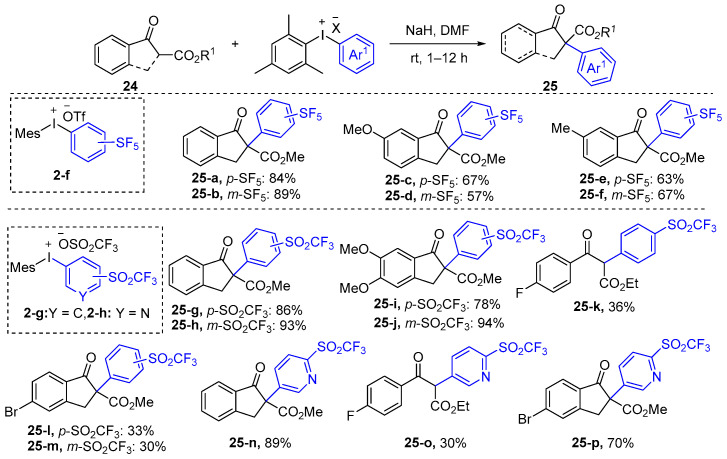
α-Arylation with SF_5_-aryl-λ^3^-iodanes and SO_2_CF_3_-containing aryl/pyridyl groups.

Subsequently, the group [68] developed a one-pot synthesis of unsymmetrical SF_5_-aryl-λ^3^-iodanes (**2-g**), employing them for SF_5_-arylation of 1,3-dicarbonyl compounds (**24**) under mild conditions (NaH/DMF) to yield SF_5_-functionalized derivatives (**25-a–25-l**). This methodology was extended to the arylation of diverse nucleophiles (e.g., phenols, anilines, carboxylates) using base or Cu catalysts. In 2017, the same group [69] synthesized SO_2_CF_3_-aryl/pyridyl iodonium salts (**2-h**), enabling trifluoromethanesulfonylation of C-/heteroatom-nucleophiles and trifluoromethanesulfonylpyridylation under some conditions, affording α-aryl/pyridyl trifluoromethanesulfonyl ketones in high yields (Scheme 13). Collectively, these approaches address regioselectivity challenges in unsymmetrical diaryliodonium chemistry and provide efficient routes to SF_5_- and SO_2_CF_3_-containing aryl/heteroaryl scaffolds—valuable precursors for bioactive compounds, catalysts, and chiral molecules.

**Scheme 14:**
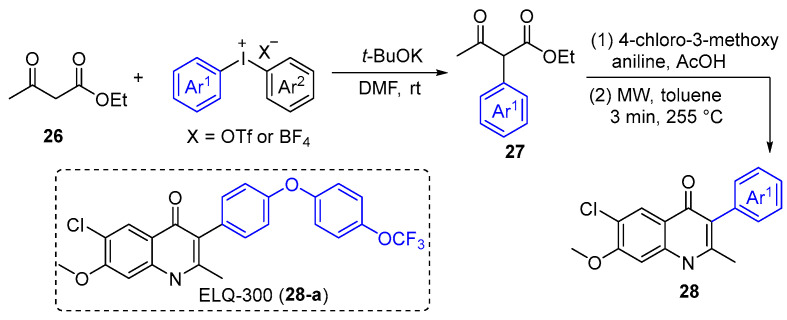
α-Arylation of ethyl acetoacetate and synthesis of ELQ-300.

In 2015, Manetsch and co-workers [70] demonstrated metal-free α-arylation of ethyl acetoacetate (**26**) using diaryliodonium triflates under ambient conditions. Whereas electron-donating/withdrawing substituents (excluding 4-methoxy groups) delivered moderate yields of α-arylated adducts, sterically encumbered systems (e.g., mesityl iodonium salts) proved substantially less competent. This methodology enabled the key arylation step in the synthesis of the antimalarial lead compound ELQ-300 (**28-a**), further demonstrating the promising utility of diaryliodonium salts as arylating agents (Scheme 14).

**Scheme 15:**
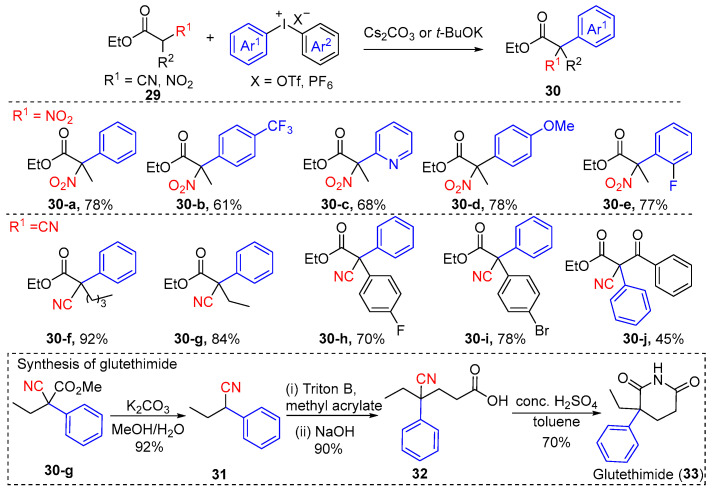
α-Arylation of α-nitro esters and ethyl 2-cyanobutanoate derivatives.

Subsequently, researchers expanded the substrate scope from dicarbonyl compounds to monocarbonyl compounds bearing ester groups with electron-withdrawing groups at the α-position. In 2015, Olofsson’s group [71] developed an efficient, direct, and metal-free approach to achieve the α-arylation of α-nitro esters. This transformation requires stoichiometric amounts of nitro esters and diaryliodonium salts, affording α-substituted nitro esters (**30-a**–**30-e**) in moderate-to-good yields with broad functional group tolerance. In 2016, Han and Wang et al. [72] developed a transition metal-free direct arylation of 2-substituted cyanoacetates with diaryliodonium salts. The synthetic methodology was successfully extended to substrates bearing diverse functional groups, enabling the preparation of various 2-substituted α-arylacetonitriles (**30-f**–**30-j**) in good yields. These compounds serve as valuable chemical intermediates for medicinal research. Significantly, the effective application of this method afforded streamlined access to glutethimide (**33**), a therapeutically relevant compound exhibiting neuroparalytic, immunomodulatory, and anticancer activities—thereby demonstrating its strategic utility in complex molecule synthesis. (Scheme 15).

**Scheme 16:**
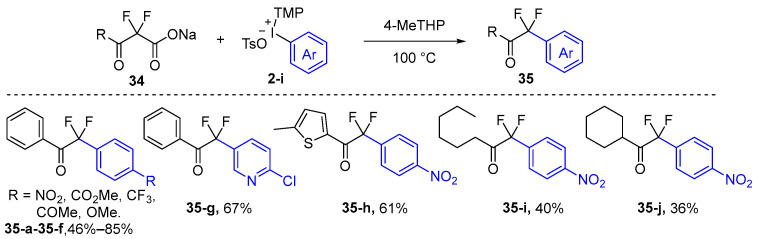
Synthesis of fluorene and spirofluorene derivatives via cyclic diaryliodonium salts.

In 2023, Kikushima et al. [73] reported catalyst-free decarboxylative arylation reaction employing α, α-difluoro-β-ketoesters (**34**) as substrates and diaryliodonium salts (**2-i**) as arylating reagents. This reaction can be applied to the synthesis of diverse α, α-difluoromethyl ketone derivatives (**35**) (Scheme 16). The synthetic significance of this reaction is twofold: it not only resides in the capacity of the α,α-difluoromethyl ketone group within the synthesized products to be converted into esters, amides, and difluoromethyl motifs prevalent in biologically active compounds, but concurrently establishes an environmentally benign methodology for constructing fluorinated pharmaceuticals and functional molecules.

### 2.3. α-Arylation of Silyl Enol Ethers

Due to silyl enol ethers being more nucleophilic than ketones, the arylation of silyl enol ethers derivatives is a strategic C–C bond-forming process that has found widespread application in organic synthesis. Over the past few decades, the diaryliodonium salts have facilitated major breakthroughs in enolate α-arylation.

**Scheme 17:**
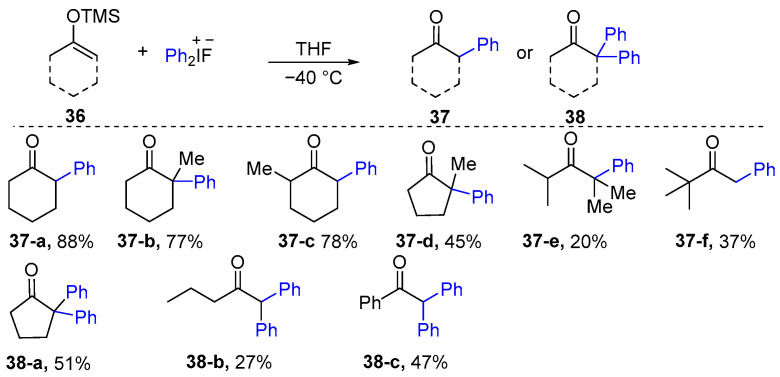
α-Phenylation of silyl enol ethers.

Koser’s group [74] investigated the α-phenylation of silyl enol ethers (**36**) using fluorinated diphenyliodonium salts (DIF). This reaction afforded α-phenyl ketones (**37**) or α, α-diphenyl ketones (**38**) in yields ranging from 20% to 88% (Scheme 17). During this study, the group examined the influence of kinetic and thermodynamic factors on regioselectivity. Their findings demonstrated that regioselectivity in α-phenylation can be controlled through appropriate condition selection.

**Scheme 18:**
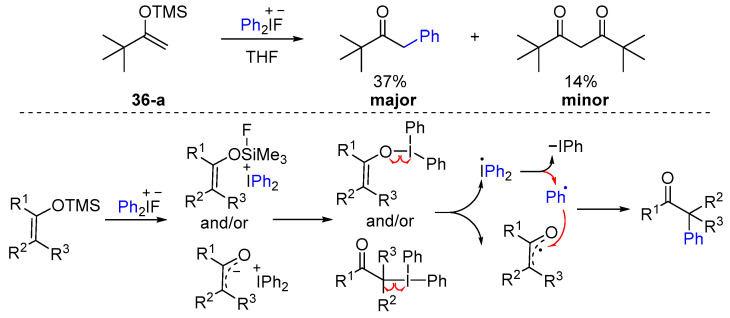
The mechanisms of α-phenylation of silyl enol ethers.

When this group subjected 3,3-dimethyl-2-(silyloxy)-1-butene (**37-f**) to DIF, the reaction not only generated the α-phenylated product (**major**) but also afforded a dehydrogenated dimeric byproduct of the ketone (**minor**) (Scheme 18). Based on experimental evidence suggesting a radical pathway, the research group proposed a plausible mechanism for this reaction.

**Scheme 19:**
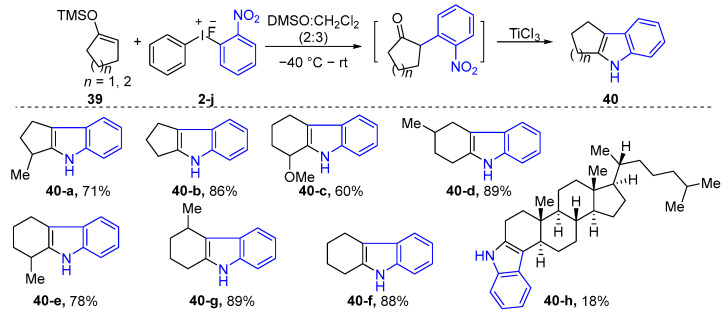
Synthesis of carbocyclic-thickened indoles.

In 1999, Rawal and co-workers [75] developed a controlled synthetic approach for carbocyclic-fused indoles (Scheme 19). This strategy commenced with the regioselective α-arylation of a silyl enol ether (**38**) using an *o*-nitrobenzene-substituted phenyliodonium salt (NPIF, **2-j**). Subsequently, the resulting nitro compound underwent TiCl_3_-mediated reduction. The generated aniline intermediate then spontaneously condensed with the adjacent ketone functionality. This efficient two-step sequence ultimately afforded a series of indole derivatives (**39**).

**Scheme 20:**
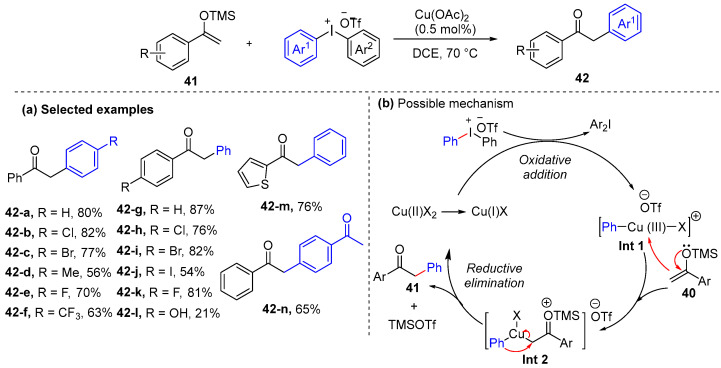
α-Arylation of arylketone-derived enolsilanes and possible mechanism.

In 2021, Taillefer and co-workers [76] reported a ligand-free and base-free Cu-catalyzed protocol for the α-arylation of arylketone-derived enolsilanes (Scheme 20). This methodology employed ketone-derived silyl enol ethers (**41**) as substrates paired with symmetric diaryliodonium salts or unsymmetric mesityl(phenyl)iodonium salts as arylating agents, achieving intermolecular C–C coupling under mild conditions, affording a series of aromatic ketone derivatives. (**42**) Critically, the ligand-free and base-free system exhibited broad functional group tolerance, accommodating sensitive moieties such as triflates and iodides (Scheme 20a).

In this case, the authors proposed the following plausible reaction mechanism (Scheme 20b): First, a Cu(III) species (**Int 1**) is generated via oxidative addition of Cu(I) into the Ar–I bond of the diaryliodonium salt. Subsequently, the key intermediate (**Int 1**) reacts with the silyl ether to afford a mixed Cu(III) aryl–alkyl complex (**Int 2**). This intermediate undergoes reductive elimination to regenerate the Cu(I) catalytic species, while simultaneously releasing the α-aryl ketone product after desilylation, mediated by the triflate anion.

### 2.4. α-Arylation of Amides

**Scheme 21:**
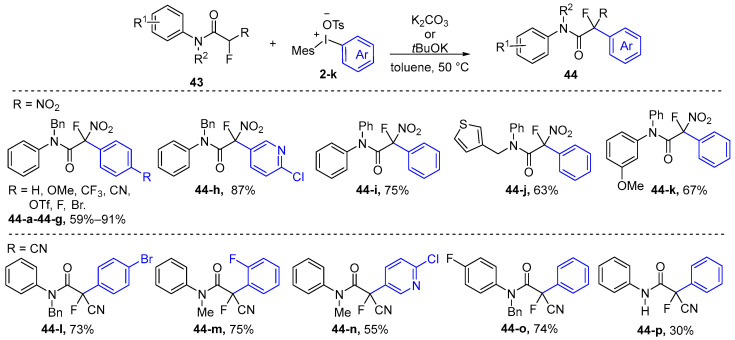
α-Arylation of α-nitro-fluoroacetamides and α-cyano-α-fluoroacetamides.

In 2020, Mohanan’s research group [77] reported a metal-free, mild, and efficient α-arylation reaction between diaryliodonium salts and α-nitro-fluoroacetamides (**43**) (Scheme 21). This reaction successfully utilized a broad range of diaryliodonium salts bearing sensitive functional groups and α-nitro-fluoroacetamides to synthesize α-arylated α-nitro-fluoroacetamide derivatives (**44-a**–**44-k**) in moderate-to-high yields. These compounds exhibit unique potential value due to their benzyl-fluorinated quaternary carbon stereocenters. Subsequently, the authors extended this methodology to α-cyano-α-fluoroacetamides with diaryliodonium salt under briefly optimized conditions, affording a series of α-aryl-α-cyano-α-fluoroacetamide derivatives (**44-l**–**44-p**). This approach exhibited excellent functional group tolerance and broad substrate scope, further highlighting the synthetic utility of the method.

**Scheme 22:**
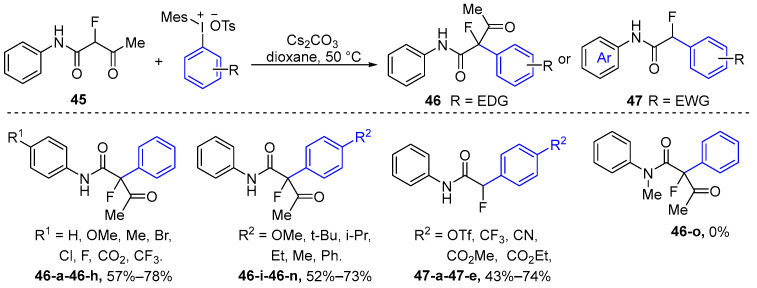
α-Arylation of α-aceto-fluoroacetamide.

Concurrently, Taillefer’s group [78] reported a metal-free methodology for synthesizing α-arylated α-fluoroacetamides (Scheme 22). This approach leveraged unsymmetric diaryliodonium salts to arylate α-aceto-α-fluoroacetamide (**45**), affording tetrasubstituted α-aceto-α-fluoroacetate derivatives (**46**) in good yields. Notably, electron-deficient diaryliodonium salts triggered a spontaneous arylation/deacylation cascade, directly yielding α-arylated-α-fluoroacetamides (**47**). The protocol’s versatility was further demonstrated through a sequential base-mediated deacylation of arylated intermediates (**46**), enabling efficient access to diverse α-aryl-fluoroacetamides (**47**). This work significantly broadened the synthetic utility of unsymmetric diaryliodonium salts as modular arylating agents.

**Scheme 23:**
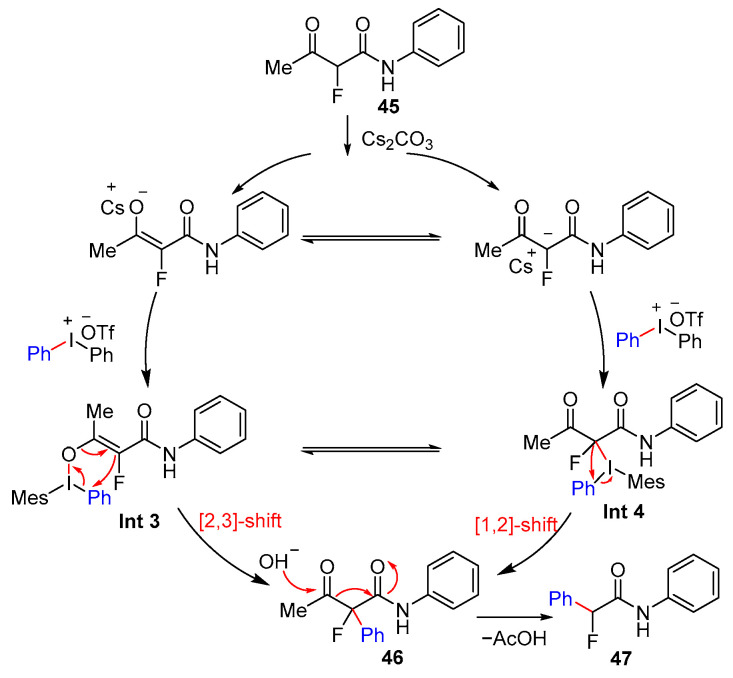
Base-promoted reaction mechanism for the α-arylation of α-fluoroacetamide.

The authors proposed a plausible mechanism (Scheme 23): Deprotonation under basic conditions generates an enolate intermediate, which subsequently coordinates with the diaryliodonium salt via C–I or O–I bond formation to afford tricoordinated iodonium intermediates **Int 3** and **Int 4**. These intermediates rapidly equilibrate, followed by characteristics [1,2]-ligand coupling or [2,3]-rearrangement to yield the arylated product (**46**). Finally, the electron-deficient aryl group enhances the electrophilicity of the fluorinated quaternary carbon center, facilitating base-mediated diacylation to deliver the deacylated product (**47**).

## 3. The Asymmetric α-Arylation of Carbonyl Compounds

The catalytic asymmetric synthesis of α-aryl carbonyl compounds has long posed a significant challenge in asymmetric catalysis. The core difficulty stems from the frequent requirement to construct a quaternary carbon stereocenter or the propensity for facile racemization at the newly formed α-aryl α-carbonyl-substituted secondary carbon stereocenter. Despite these inherent challenges, the past two decades have seen the development of diverse innovative strategies for the catalytic asymmetric α-arylation of racemic carbonyl compounds. Among these approaches, the catalytic enantioselective α-arylation employing diaryliodonium salts as arylating agents stands out as a particularly efficient and robust method. This strategy provides a streamlined route to enantioenriched α-aryl carbonyl compounds—structurally important motifs with substantial synthetic value. In the following, we will introduce the related progress on the basis of the diaryliodonium salts as arylating agents employed.

**Scheme 24:**
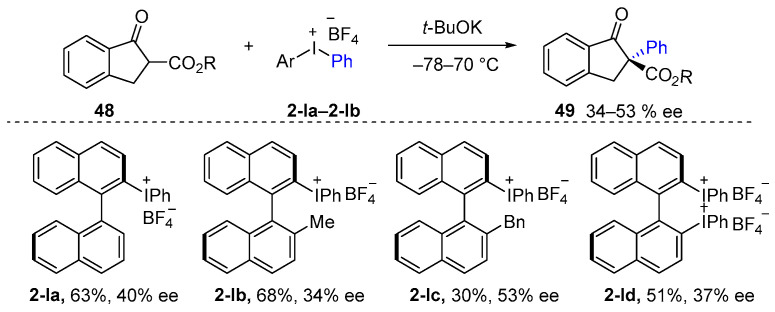
Asymmetric α-phenylation involving chiral diaryliodonium salts.

In 1999, Ochiai et al. [79] pioneered the synthesis of chiral diaryliodonium salts (**2-l**) via a BF_3_·Et_2_O-catalyzed tin-λ^3^-iodane exchange. Concurrently, the group developed an asymmetric α-phenylation of cyclic β-ketoesters (**48**) employing a chiral binaphthyl(phenyl)iodonium salt as the aryl source. This transformation afforded the corresponding products (**49**) in 34–53% yield with enantioselectivities ranging from 34% to 53% ee. This work highlighted the feasibility of asymmetric α-arylation using chiral diaryliodonium salts. Although reports on the asymmetric synthesis of chiral diaryliodonium salts remain scarce, this strategy provides a novel approach for asymmetric α-phenylation and significantly expands the synthetic utility of diaryliodonium salts (Scheme 24).

**Scheme 25:**
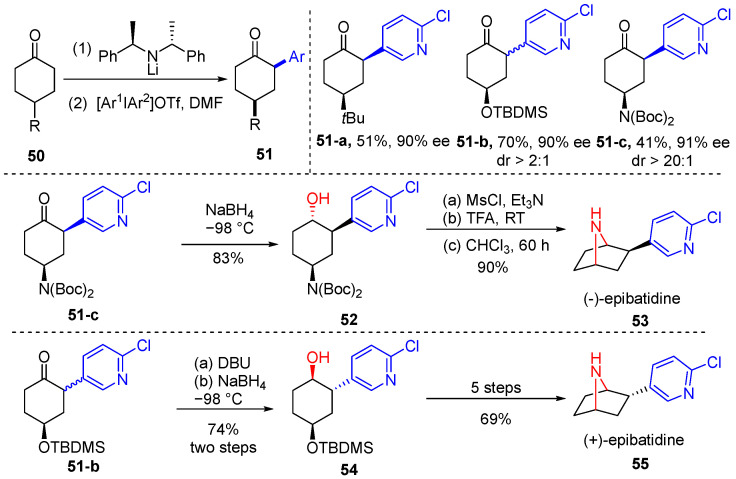
Simpkins’ base-mediated asymmetric α-arylation.

In 2005, Olofsson’s group [80] reported a direct asymmetric α-arylation of cyclohexanones. This method involves the asymmetric enolization of 4-substituted cyclohexanones (**50**) using Simpkins’ base, followed by coupling with diaryliodonium salts to afford α-aryl ketones (**51**) in moderate-to-good yields with high enantioselectivity. Notably, this methodology was further applied to the short and efficient total synthesis of (-)-epibatidine (**53**) and the formal synthesis of (+)-epibatidine (**55**) (Scheme 25).

**Scheme 26:**
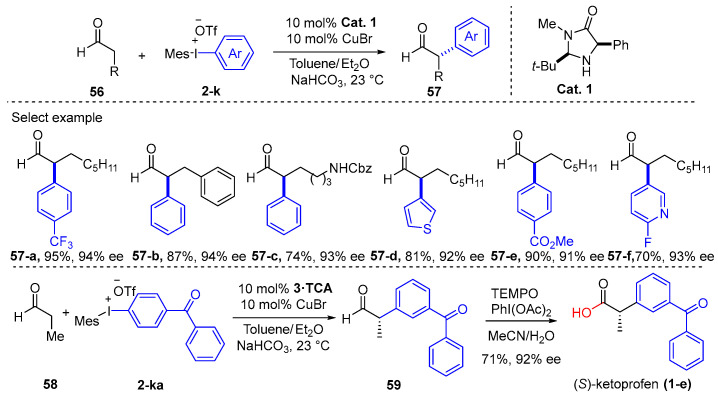
Enantioselective α-arylation catalyzed by chiral amine catalyst TCA.

In 2011, MacMillan and co-workers [81] disclosed an enantioselective α-arylation of unactivated aldehydes (**56**) employing diaryliodonium salts as arylating agents. This transformation was achieved through a dual catalytic system comprising chiral amine catalyst TCA (**Cat. 1**, 10 mol%) and CuBr (10 mol%). This method achieved excellent yields (67–95%) and outstanding enantioselectivity (91–94% ee), delivering a series of α-chiral tertiary carbon-containing esters (**57**). These mild catalytic conditions provided a novel strategy for the enantioselective construction and retention of stereocenters at the enolizable α-formyl benzylic position. Furthermore, this group successfully applied this methodology as a key step to the rapid synthesis of (*S*)-ketoprofen (**1-e**), an oral topical analgesic (Scheme 26).

**Scheme 27:**
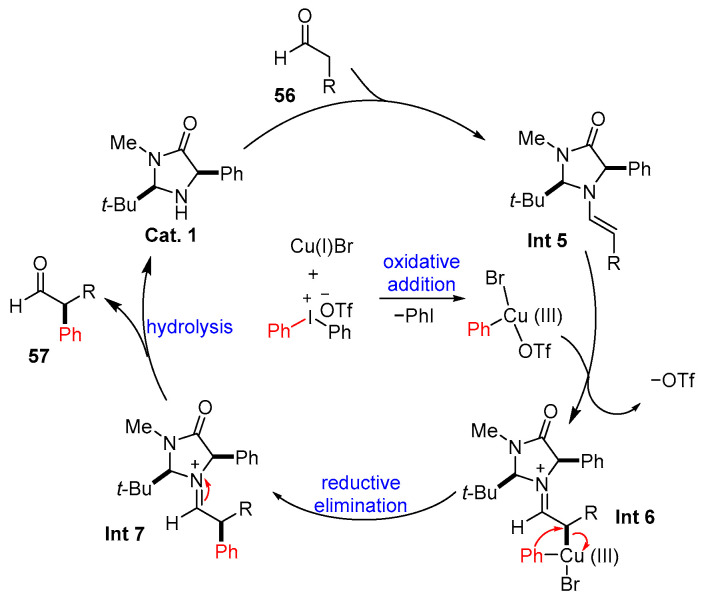
Catalytic mechanism of the chiral amine catalyst TCA.

Building on this work, the group further explored the reaction mechanism (Scheme 27). They suggested that this tandem catalytic pathway starts with the condensation of amine catalyst (**Cat. 1**) with aldehyde (**56**) substrate to form activated chiral enamine **Int 5**, followed by the oxidative addition of Cu(I) to the C-I bond of diaryliodonium triflate system to form electron-deficient aryl Cu(III) species, which is then followed by a highly electrophilic Cu(III) aryl system that will be rapidly coordinated with the *Re* face of the chiral enamine to form the π-Cu complex (**Int 6**). After which, rapid bond isomerization occurs to form the η1-imino **Int 7**, which is eliminated by reduction to release the optically enriched α-site aryl imine, rebuild the Cu(I)Br catalyst, and hydrolyze the imine to give the desired α-site aryl aldehyde product (**57**), whilst allowing the organocatalyst to be released and participate in the next cycle.

**Scheme 28:**
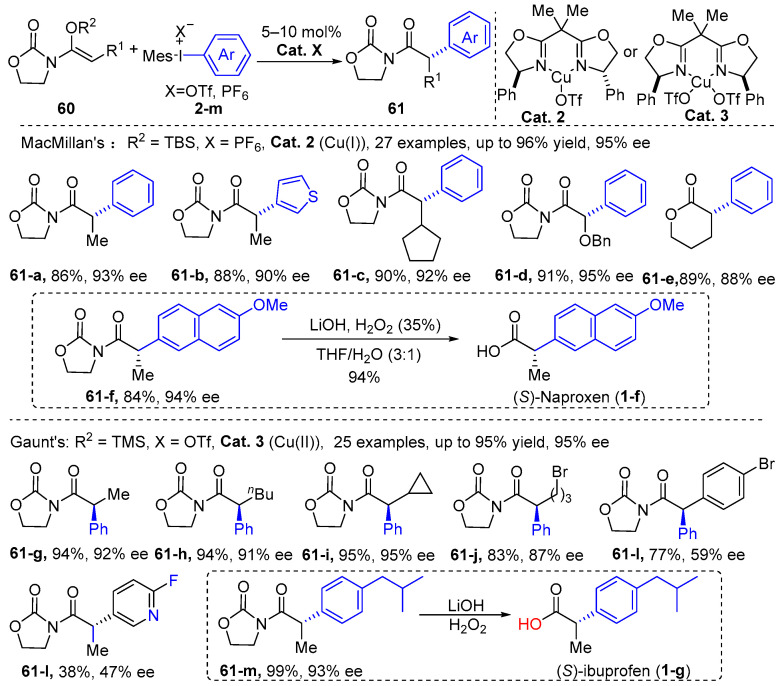
Asymmetric α-arylation catalyzed by a chiral Cu(II)-bis(oxazoline) complex.

In a nearly concurrent publication, MacMillan’s group [82] reported a copper-catalyzed asymmetric α-arylation reaction involving diaryliodonium salts. This reaction employs a preformed Cu complex (**Cat. 2**, derived from (*S*, *S*)-Ph-Box and CuOTf, 10 mol%), to achieve arylation of *N*-acyl oxazolidinones (**60**) derived from lactones or acyl oxazolidinones with diaryliodonium hexafluorophosphates, delivering arylative products (**61**). This reaction provided the arylative products in good-to-excellent yields (65–96%) and high enantioselectivity (87–95% ee) under mild conditions, offering a novel strategy for constructing stereocenters at enolizable α-carbonyl benzylic positions. To demonstrate its utility, the group applied this approach to the rapid enantioselective synthesis of the NSAID (S)-naproxen (**1-f**). Concurrently, Gaunt et al. [83] reported the arylation of *N*-acyl oxazolidinones (**60**) using a chiral Cu(II)-bis(oxazoline) (**Cat. 3**) catalytic system. Similarly effective under mild conditions, their method afforded excellent yields and enantioselectivity, delivering valuable building blocks (**61-g–61-m**) and enabling the synthesis of important NSAIDs and analogs. Notably, they achieved gram-scale synthesis of ibuprofen (**1-g**) with 99% yield and 93% ee (Scheme 28).

**Scheme 29:**
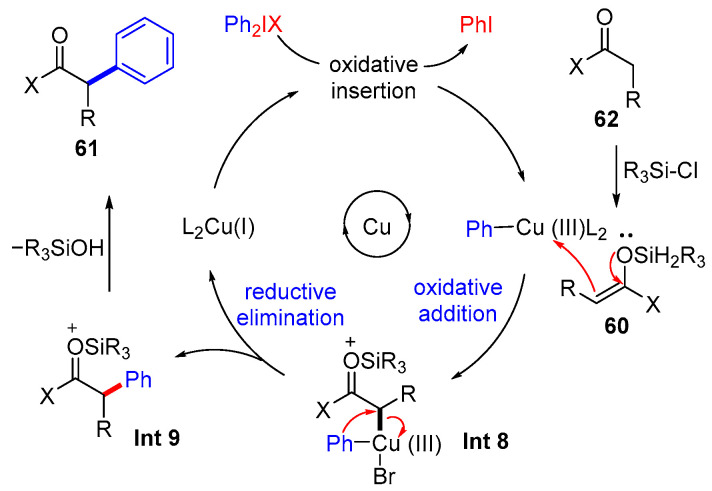
Mechanism of cooperative catalysis reaction by ligand-bound Cu(I) complex.

They proposed the following mechanism (Scheme 29): The ligand-bound Cu(I) complex undergoes oxidative insertion into the diaryliodonium salt, generating a highly electrophilic chiral Cu (III) species (**Int 8**). This intermediate is captured by the silyl enol ether nucleophile (**60**), followed by reductive elimination and hydrolysis of the silyl group (**Int 9**) to yield the desired α-aryl carbonyl product (**61**), while the Cu(I) catalyst is regenerated to participate in the next catalytic cycle.

**Scheme 30:**
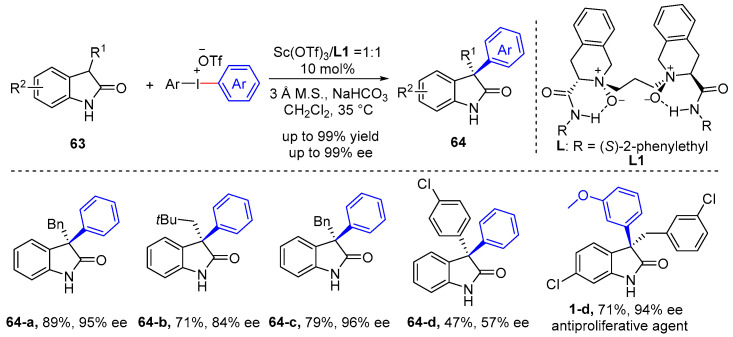
Enantioselective α-Arylation of *N*-unprotected 3-substituted oxindoles.

In 2013, Feng and co-workers [84] demonstrated that chiral *N*, *N*′-dioxide-derived metal complexes also serve as effective catalysts for the enantioselective α-arylation of carbonyl enolates with electrophilic diaryliodonium salts (Scheme 30). Under the presence of NaHCO_3_, the combination of 10 mol% chiral *N*, *N*′-dioxide (**L1**), and 10 mol% Sc (OTf)_3_ complexes as a chiral Lewis acid efficiently enabled the enantioselective α-arylation of *N*-unprotected 3-substituted oxindoles (**63**) with diaryliodonium triflates. This reaction afforded oxindole derivatives bearing quaternary carbon centers (**64**) in good-to-excellent yields (up to 99%) and high enantioselectivity (up to 99% ee). Notably, this methodology was successfully applied to the enantioselective synthesis of an antiproliferative agent (**1-d**) for cancer treatment.

**Scheme 31:**
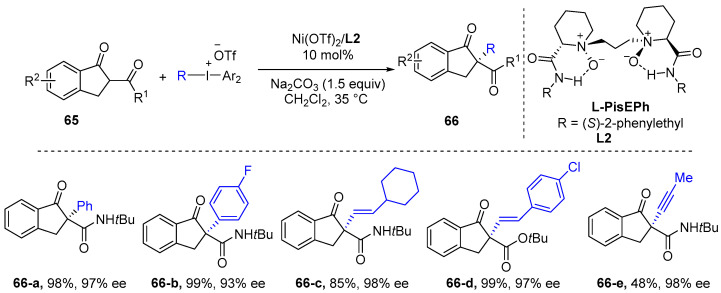
Enantioselective α-arylation/vinylation/alkynylation of cyclic β-keto amides/esters.

Three years later, this research group [85] achieved highly enantioselective α-arylation/vinylation/alkynylation of cyclic β-keto amides/esters (**65**) with diaryliodonium triflates, using a chiral *N*, *N*′-dioxide (**L2**)/Ni (OTf)_2_ complex as the catalyst. Under optimized conditions (10 mol% catalyst loading), a series of α-aryl/α-vinyl/α-alkynyl-substituted β-keto amides/esters (**66**) were synthesized with yields and enantiomeric excess (ee) values reaching up to 99% (Scheme 31).

**Scheme 32:**
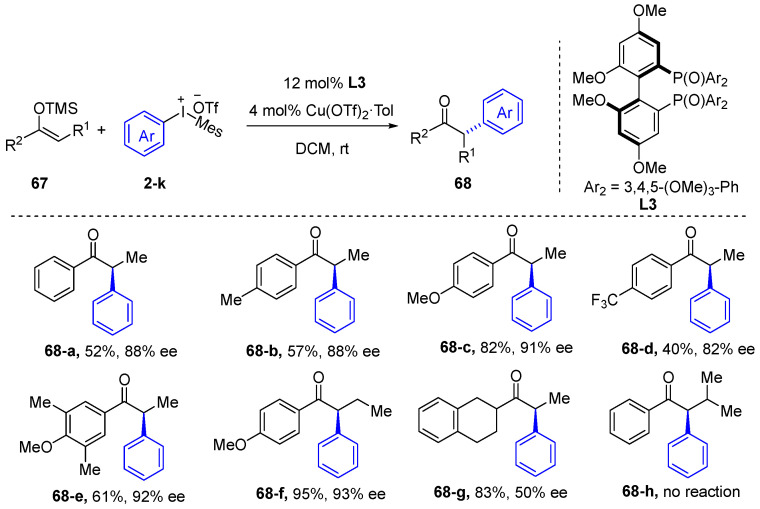
Enantioselective α-arylation of acyclic ketones.

In 2021, Orlandi’s group [86] discovered a novel catalytic system based on Cu(II) and a chiral bisphosphine dioxide ligand (**L3**), successfully achieving the asymmetric α-arylation of acyclic ketones at the carbonyl α-position (Scheme 32). Under mild catalytic conditions with 12 mol% of the chiral bisphosphine dioxide ligand and 4 mol% Cu(OTf)_2_·Tol, the reaction utilized silyl enol ethers of acyclic ketones (**67**) as substrates and diaryliodonium salts as arylating agents, delivering a series of α-chiral tertiary carbon-containing carbonyl compounds (**68**) in excellent yields with high enantioselectivity. The experimental results showed that the silyl enol ethers bearing electron-donating substituents in *meta*- or *para*-positions were found to give lower selectivity, likely due to the geometrical bias provided by the *ortho*-OMe group. Additionally, the steric hindrance next to the reaction site was detrimental, such as *i*Pr or Bn groups (**68-h**), onto the nucleophilic α-carbon.

Up to date, the catalytic asymmetric α-arylation of carbonyl compounds using diaryliodonium salts have remarkably developed in the past two decades, this field of research is still in its early stages and daunting challenge. The research groups’ results showed that the substrates bearing electron-donating substituents in *meta*- or *para*-positions were found to give lower enantioselectivity, likely due to the geometrical bias provided by the *ortho*-OMe group (Table 1, Entry 3). Additionally, the steric hindrance next to the reaction site was detrimental, such as *i*Pr, Bn groups, etc. (Table 1, Entry 2,4), and diaryliodonium salts featuring the *ortho*-positions were found to give lower reactivity (Table 1, Entry 4).

**Scheme 33:**
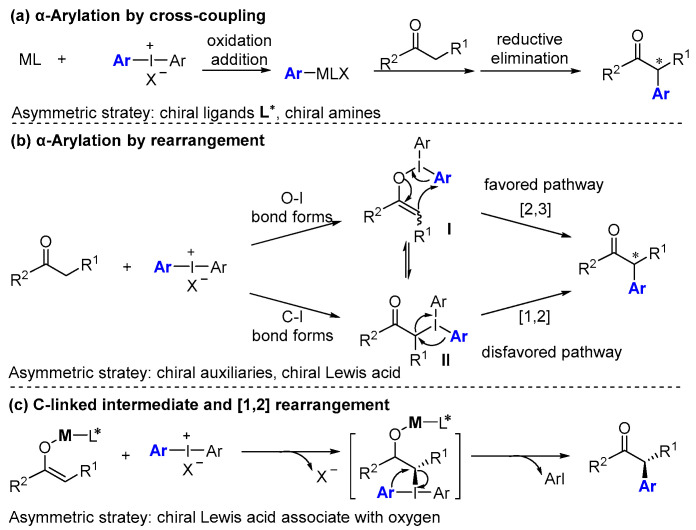
Possible mechanism for α-arylation of carbonyl compounds.

Based on the above asymmetric α-arylation reaction protocols. Scheme 33 summarizes the several possible mechanisms for α-arylation of carbonyl compounds reported so far. For example, (a) α-Arylation by cross-coupling, (b) α-arylation by rearrangement, and (c) C-linked intermediate and [1,2] rearrangement.

Currently, diaryliodonium salts are generally believed to react by the reductive elimination pathway, delivering the equatorial aryl moiety to the axially installed nucleophile (Scheme 33a). Furthermore, the involvement of electrophilic addition and aryl moiety rearrangement was also proposed. Olofsson and co-workers, through computational study, confirmed that the addition of diaryliodonium salts to the enolate results in almost isoenergetic oxygen–iodine- and carbon–iodine-bonded isomers and that reaction could follow associative or dissociative ligand-exchange pathways. The [2,3]-rearrangement pathway was favored over the [1,2] process (Scheme 33b) [87]. Nevertheless, Feng and co-workers [84] proposed that by introducing chiral Lewis acids with strong oxygen affinity, C-linked enantiomeric intermediates could be formed, which could then undergo [1,2] rearrangement to achieve asymmetric arylation (Scheme 33c). The proposal of this mechanism has important implications for the development of arylation reactions involving non-oxidative metal participation or organocatalysis.

## 4. Summary and Outlook

In summary, the great advantages of diaryliodonium salts, for example, with their low toxicity compared with heavy-metal reagents, mild reaction conditions, fast accessibility of a large variety of reagents, and easy handling, have established themselves as versatile arylation reagents in organic synthesis over recent decades. Their exceptional reactivity has driven significant progress in α-arylation strategies for carbonyl compounds, enabling transformations under mild conditions with remarkable functional group tolerance and regioselectivity. Furthermore, advancements in chiral ligand design, organocatalysis, and asymmetric catalytic systems have unlocked unprecedented potential for enantioselective α-arylation reactions using these iodonium salts. In spite of the remarkable achievements to date, this field of research is still in its early stages and brimming with potential opportunities for further synthetic exploration. For example: (a) The reaction mechanism for the arylation of diaryliodonium salts is worthy of further exploration [87]. (b) Chemoselectivity and regioselective migration in unsymmetric diaryliodonium salt reactions remains a critical challenge [66,88,89]. (c) The reaction involving diaryliodonium salts fails to meet the atom economy requirement, as only one aryl group undergoes productive coupling while the counterpart is converted into stoichiometric aryl iodide byproducts. This inherent stoichiometric inefficiency underscores the ongoing challenge of developing methodologies for dual-aryl utilization of these reagents [90,91,92,93,94]. (d) The current focus of synthetic applications lies in the preparation of bioactive compounds using diaryliodonium salts, and the total synthesis of natural products and functional materials using diaryliodonium salts as arylating agents is rarely reported. Thus, the further expansion of diaryliodonium salts as arylating agents in the preparation of the above-mentioned compounds is needed to be explored, especially in practical applications [63,95]. (e) The development of novel chiral ligands, organocatalysts, and chiral catalyst systems for the enantioselective α-arylation reactions of carbonyls and related compounds will persist as a pivotal research frontier. This is because the diverse access to optically active α-aryl carbonyl molecules will continue to be explored, and such exploration will be beneficial for the discovery and development of related drugs.

## Figures and Tables

**Table 1:** Synthetic protocols and strategy for enantioselective α-arylation of carbonyl compounds.

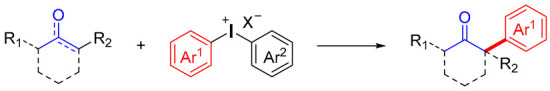								
Entry	Substrate	Catalyst Systems	Enantioselectivity	Efficiency	Functional Group Tolerance		Substrate Scope	Ref.
					Substrate	Diaryliodonium Salts		
1	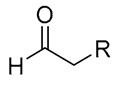	Combination of copper and organic catalysts (**Cat. 3·TCA** and CuBr)	90–94% ee	**Cat. 3·TCA** (10–40 mol%) CuBr (5–10 mol%) NaHCO_3_, 23 °C 67–95% yield	CO_2_Et, NBoc, NHCbz, Alkene, etc.	F, CF_3_, OMe, NO_2_, etc.	R = Alkyl, *i*Pr, Bn; Ar^1^ = Aryl, Thiophene, Pyridine, Naphthalene group, etc.	[81]
2	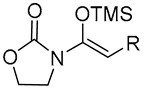	Cu(II)-bis(oxazoline)	17–94% ee	**Cat. 3** (5–10 mol%), 0–10 °C, 6–20 h, 40–99% yield	NMeCbz, Alkene, etc.	CO_2_Et, F, CF_3_, OMe, etc.	R = Alkyl, Aryl, Indole, *i*Pr, Bn; Ar^1^ = Aryl, Thiophene, Naphthalene group, etc.	[82]
3			90–94% ee	**Cat. 4** (10–20 mol%), −20–0 °C, 24 h 65–96% yield	NMeCbz, Alkene, etc.	CO_2_Et, F, CF_3_, OMe, etc.	R = Alkyl, Indole, *i*Pr, Bn; Ar^1^ = Aryl, Thiophene, Naphthalene group, etc.	[83]
4	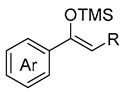	Cu(I)− Bis(phosphine) Dioxide	45–95% ee	**L3** (12 mol%) Cu(OTf)_2_·Tol (4 mol%) rt, 16 h 20–90% yield	NCbz, F, CF_3_, OMe, NO_2_, etc.	CO_2_Et, F, Cl, Br, CF_3_, OMe, NO_2_, etc.	R = Alkyl, *i*Pr, Bn, Tetralone; Ar^1^ = Aryl, Pyridine group, etc.	[86]
5	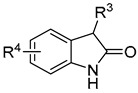	N,N′-dioxide-Sc(OTf)_3_ complex	17–99% ee	Sc(OTf)_3_/**L1** =1:1 10 mol%, 3 Å M.S., NaHCO_3_, 35 °C 35 °C, 24–120 h 47–99% yield	F, Cl, Br, CN, OMe, NO_2_, etc.	F, Cl, Br, Ph, Me, etc.	R = Alkyl, 2-naphthylmethyl; Ar^1^ = Aryl, Pyridine group, etc.	[84]
6	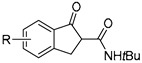	N,N′-dioxide (**L2**)/Ni(OTf)_2_ complex	91–99% ee	L-PisEPh/Ni(OTf)_2_ (1:1.2; 10 mol %) Na_2_CO_3_, 35 °C, 24–120 h 48–99% yield	F, Cl, Br, Ph, etc.	F, Cl, Br, Ph, Me, etc.	Ar^1^ = Aryl group	[85]

## Data Availability

No new data were created or analyzed in this study.
